# Operative Surgery

**Published:** 1903-09

**Authors:** 


**Operative Surgery.** By HERBERT WILLIAM ALLINGHAM. Pp. xiv., 367. London: Baillière, Tindall, and Cox. 1903.

This book is intended for "surgeons wishing to hastily look up the leading features of an operation, and also to those who are performing operations on the cadaver." We are aware of the difficulties in carrying out this intention, but a perusal of the book leaves some dissatisfaction from both points of view. The illustrations number over 200, and are generally good and clear, but fig. 196 is anatomically incorrect in several particulars, and fig. 199 does not correspond with the description in the text. There are several grammatical and clerical errors, *e.g.*, "jackanet," catgut in "1 in 500 carbolic solution," the "innermost" of two white lines, "Parker Symes" (Syms), and "either . . . are" (p. 357). On page 10 the flexor carpi radialis is stated to be on the inner side of the front of the wrist. Concerning the subject matter, in excising the clavicle, it is stated that the periosteum should be stripped off; and in excising the maxilla with preliminary ligature of the external carotid it would seem superfluous to perform tracheotomy and plug the pharynx as recommended. Tenotomy of the tendo-achillis is thus described (p. 95): "The patient being on his back, the foot is rotated outwards. The assistant keeping the tendon on the stretch, the knife (*sic*) is entered about an inch above the os calcis, on the inner side, and the tendon divided." The removal of half the tongue is described as accomplished by clamping the base of the tongue and ligating it in mass—a clumsy, and, we think, dangerous procedure. The horizontal incision along the clavicle in thyroidectomy (fig. 127) might be taken exception to, and also to the absence of any mention of the division of palatal muscles in staphylorrhaphy, the method of dealing with an irreducible intussusception, and the scanty description of nephrorrhaphy. We would call attention to the excellent method of subcostal opening of the pericardium described on page 222. Otherwise the book is readable, of convenient size, and will prove a useful addition to works of its class.

